# Shear stress improves the endothelial progenitor cell function via the CXCR7/ERK pathway axis in the coronary artery disease cases

**DOI:** 10.1186/s12872-020-01681-0

**Published:** 2020-09-07

**Authors:** Hua Zhou, Qiang Tu, Yan Zhang, Hua Qiang Xie, Qing Yun Shuai, Xiao Chuan Huang, Jie Fu, Zheng Cao

**Affiliations:** 1 Department of Medical Ultrasound, Taihe Hospital, Hubei University of Medicine, Shiyan, 442000 Hubei China; 2 Department of Cardiology, Taihe Hospital, Hubei University of Medicine, Shiyan, 442000 Hubei China; 3 Hubei Key Laboratory of Embryonic Stem Cell Research, Taihe Hospital, Hubei University of Medicine, Shiyan, 442000 Hubei China

**Keywords:** Shear stress, Endothelial progenitor cells, Coronary artery disease

## Abstract

**Background:**

Dysfunction in the late Endothelial Progenitor Cells (EPCs) is responsible for endothelial repair in patients with Coronary Artery Disease (CAD), and the shear stress is beneficial for EPCs function. However, the impact of shear stress on the capacity of EPCs in CAD patients has not been elucidated yet. The C-X-C chemokine receptor 7/extracellular signal-regulated kinase (CXCR7)/(ERK) pathways are identified to regulate EPCs function in CAD patients. Here, we hypothesize that shear stress upregulates the CXCR7/ERK pathways, which restore the EPCs function in CAD patients.

**Methods:**

The human Peripheral Blood Mononuclear Cells (PBMCs) were collected from healthy adults and CAD patients and then used for EPCs cultivation. The Lv-siRNA for human CXCR7 was transfected into induced EPCs isolated from the CAD patients. Meanwhile, the EPCs from CAD patients were subjected to shear stress generated by a biomimetic device. Next, the cell viability, migration, tube formation, and apoptosis were detected by CCK-8, Transwell assay, Matrigel, and flow cytometry, respectively. Also, the CXCR7/ERK pathways in human EPCs were analyzed by Western blotting and qRT-PCR.

**Result:**

Compared to the EPCs collected from normal adults, the CAD patient-derived EPCs showed reduced in vitro vasculogenic capacity. Also, the level of CXCR7 in CAD patient-derived EPCs was significantly reduced compared to the EPCs of healthy subjects. Meanwhile, the extracellular signal-regulated kinase (ERK), which represents a CXCR7 downstream signaling pathway, had decreased phosphorylation level. The shear stress treatment augmented the CXCR7 expression and also elevated ERK phosphorylation, which is comparable to the up-regulation of CAD patient-derived EPCs function. Further, the small interfering RNA (siRNA)-mediated CXCR7 knockdown diminished the enhanced migration, adhesion, and tube formation capacity of shear stress treated CAD patient-derived EPCs.

**Conclusion:**

Up-regulation of the CXCR7/ERK pathways by shear stress can be a promising new target in enhancing the vasculogenic ability of CAD patient-derived EPCs.

## Background

Coronary artery disease (CAD) is one of the frequently occurring cardiovascular diseases (CVDs) across China [1–3]. Increasing evidence has revealed that CAD patients suffer from abnormal endothelial structure and function [4–7]. Thus, it is vital to maintain vascular endothelial integrity to treat CAD. The bone marrow-derived circulating endothelial progenitor cells (EPCs) play an important role in the endothelial repair process after endothelial damage is caused [8–10]. Unfortunately, the EPCs function is impaired in CAD patients delaying the progression of vascular endothelial repair response [11–13]. Diabetes-related vasculopathy is the main risk factor for aggravating cardiovascular burden because impaired insulin sensitivity disrupts the vascular redox balance and thereby contributes to endothelial dysfunction [14–16]. Thus, it is necessary to identify a new way to improve the EPCs function in CAD patients and reduce the occurrence of CVDs. Shear stress is a measure of non-pharmacological intervention which benefits the vascular endothelial homeostasis [17–19]. Nonetheless, the underlying mechanism by which shear stress affects the CAD-EPCs at the molecular level remains unclear so far. In recent years, CXC chemokine receptor 7 (CXCR7) is considered a new chemokine receptor that responds to the stromal cell-derived factor 1(SDF-1) and regulates numerous biological processes [20–22]. Based on our previous study, impaired phosphorylation of the CXCR7-mediated extracellular signal-regulated kinase (ERK) pathway is involved in CAD-related decline of EPCs function in vitro [23].

However, only little information is available to understand the relationship between shear stress and EPCs function in CAD patients. Based on the previous studies, we assumed that impairment of the vasculogenic ability of CAD-derived EPCs was associated with the CXCR7 down-regulation and reduced ERK activation. Besides, shear stress may promote the EPCs vasculogenic function in CAD patients, and this was correlated with the increase in the CXCR7/ERK signaling. Accordingly, this study aims to explore the effect of shear stress on the up-regulation of the CXCR7/ERK pathways in EPCs function of CAD patients. Our work explored the vasculogenic capacity in vitro along with the CXCR7/ERK signaling pathways in EPCs among CAD and healthy controls. Subsequently, CAD-derived EPCs were subjected to a physiological condition of shear stress using a biomimetic device and were examined in vitro to test their effects on the CXCR7 signaling pathway and their vasculogenic function.

## Methods

### Characteristics of subjects

Based on the coronary angiography examination, ten consecutive male outpatients recently diagnosed with CAD (determined by≥50% stenosis), and ten normal controls with matched ages were recruited to this study. Patients with hypertension, an active inflammatory disorder, diabetes, cardiovascular events, malignancy, and additional cardiovascular risk factors, were excluded from this study. Table 1 displays the baseline characteristics of CAD patients and normal controls. Routine tests, such as BMI, HR, blood pressure, fasting plasma glucose, biochemical indexes, etc. were gauged and recorded. The study protocol was approved by the Ethics Committee of Taihe Hospital. Each participant provided the written informed consent before their participation.

**Table 1:** The baseline subject features

Group	Control (***n***=10)	CAD patients (***n***=10)	***P*** value
Age(yrs)	59.9±8.17	64.6±6.97	0.483
BMI(kg/m^2^)	22.44±1.8	23.92±2.57	0.391
HR(bpm)	70.1±7.64	72.1±8.14	0.628
Systolic blood pressure (mmHg)	117.4±11.4	118±10.8	0.97
Diastolic blood pressure (mmHg)	76.7±6.55	77.7±6.86	0.873
Fasting plasma glucose (mmol/L)	4.82±0.37	4.91±0.49	0.519
Total cholesterol (mmol/L)	4.38±0.58	4.49±0.62	0.5
Triglicerides(mmol/L)	1.53±0.1	1.48±0.68	0.335
LDL cholesterol(mmol/L)	3.11±0.34	3.33±0.22	0.615
HDL cholesterol(mmol/L)	1.5±0.16	1.35±0.22	0.161
Blood urea nitrogen (mmol/L)	4.66±0.56	4.93±0.7	0.384
Creatinine (μmol/L)	68.6±9.52	73.6±11.2	0.303
Alanine aminotransferase (U/L)	26.7±6.07	24.6±5.74	0.56
Aspartate aminotransferase (U/L)	19.8±2.04	21.6±5.81	0.068

Data are shown as mean ± SD

CAD coronary artery disease, *BMI* body mass index, *HR* heart rate, *HDL* high-density lipoprotein, *LDL* low density lipoprotein

### Culture of late EPCs (LEPC)

The LEPCs were isolated and cultured according to the previous description [24]. The human-derived peripheral blood mononuclear cells (PBMCs) were inoculated into 6-well plates containing 5 μg/mL human fibronectin, followed by culturing in the EGM-2 medium (Promocell, USA). The cells were cultured at 37 °C with 5% CO_2_ in a humid environment. After 4 days of culture, the cells were washed with PBS to remove the non-adherent cells, and the original medium was replaced. Typically, EPCs were positive in the uptake of 0.02 mg/mL DiI-acLDL (Invitrogen, USA) and binding of 0.01 mg/mL FITC-conjugated BS-1 lectin (Sigma-Aldrich, USA), according to the previous description [25]. Also, flow cytometry was done to examine the endothelial markers in the cultivated LEPCs using the following antibodies: 1. FITC-conjugated monoclonal mouse anti-human antibodies that recognize CD31 (eBioscience, USA) and vWF (Novus Biologicals, USA), and 2. APC-conjugated monoclonal mouse anti-human antibodies that recognize Tie-2 (Novus Biologicals, USA). Notably, LEPCs at the third passage (approximately 4 weeks) were utilized in each of the assays. According to the separation and culture protocol, EPCs were the cells that attached and appeared spindle-shaped [26].

### Shear stress test

EPCs were subjected to shear stress using the flow chamber loading device. Firstly, the EPCs were inoculated on the glass slides and subsequently put on the parallel-plate flow chamber channel. After culturing for 1 day, the hydrodynamic shear stress was applied to the adherent cells on a parallel-plate flow chamber, according to the previously depicted procedure [27]. Later, the seeded cells were exposed to 6, 12, and 24 h of shear stress at 0, 5, 15, and 30 dyn/cm^2^, separately. Typically, the shear stress was determined according to the formula, T = 6Qμ/bh stress, where Q stands for flow rate, μ is the medium viscosity, b is the channel width, and h is the channel height. While the control cells were maintained at static conditions, each experiment was carried out in an incubator at 37 °C with 5% CO_2_.

### Knockdown of CXCR7

The CXCR7 level in EPCs was knocked down using the Mission lentiviral siRNA transfection particles. Viral transfection was performed according to the manufacturer protocol (GenePharma Co., Ltd., Shanghai). After culturing the cells for 28 days, they were transferred into a serum-free medium containing the CXCR7-targeting siRNA (CXCR7-siRNA) or non-targeting siRNA (scrambled-siRNA) lentiviral particles. After 6 h of transfection, cells were washed with PBS and incubated for 48 h in the EPCs medium before analysis.

### EPCs proliferation

After culturing for 28 days, the human-derived EPCs were subjected to laminar shear stress in vitro for 6, 12, and 24 h at 0, 5, 15, and 30 dyn/cm^2^, separately. EPCs or EPCs treated with shear stress were placed on the glass slides (1 × 10^4^ cells per well) and cultured for another 6, 12, and 24 h. Then, the glass slides were placed in the 24-well plates containing 200 μL of fresh medium supplemented with 20 μL CCK8 (Beyotime Biotechnology, China) solution, followed by 1 h incubation. Then, a microplate reader (Thermo Fisher, USA) was used to determine the absorbance at a wavelength of 450 nm.

### EPCs migration test

The modified Boyden chamber (Millipore, USA) was adapted to determine the migratory capacity of EPCs. 5 × 10^4^ EPCs were resuspended into 250 μL of serum-free EBM-2 medium and pipetted onto the upper chamber. Later, this chamber was placed into a 24-well dish supplemented with 500 μL EBM-2 medium containing 10% FBS. After incubating for 8 h at 37 °C, investigators, who were blind to the treatment grouping counted the transmigrated cells independently.

### EPCs adhesion test

3 × 10^4^ EPCs were resuspended into 300 μL of EBM-2 medium and pipetted onto a 24-well plate coated with human fibronectin (5 μg/mL) and incubated at 37 °C for 3 h. Later, the 24-well plate was washed with PBS to remove the non-adherent cells, while the adherent cells were fixed with 4% paraformaldehyde and stained using 0.1% crystal violet. Finally, the investigators blind to the treatment grouping counted the adherent cells independently.

### Tube formation test for EPCs

The growth factor-depleted Matrigel (BD) was thawed overnight at 4 °C. After complete thawing, 100 μL of Matrigel was added in the 24-well plates for even distribution, followed by 2 h incubation at 37 °C. 1 × 10^5^ EPCs were then resuspended using 500 μL of EBM-2 medium and then added onto the Matrigel top. After incubating for 4 h at 37 °C, each well was imaged using the microscope, and the mean tubule number was calculated based on 3–5 randomly selected fields.

### EPCs apoptosis assay

The Annexin V-FITC Apoptosis Detection Kit (eBioscience, USA) was used to examine the apoptosis of EPCs. After 12 h of shear stress treatment, Annexin V-FITC, along with propidium iodide (PI), was added to the rinsed cells at a density of 1 × 10^6^ cells/mL in the FACS buffer at ambient temperature for 15 mins in the dark. Next, cells were analyzed using flow cytometry.

### RNA isolation and real-time qRT-PCR

The Trizol reagent (Takara, Japan) was used to extract the total RNA. The SYBR® Green PCR Master Mix and iSript cDNA Synthesis Kit (Takara, Japan) were used for real-time qRT-PCR as per the manufacturer’s instructions. The expression levels were corrected based on the GAPDH mRNA level and calculated according to the 2^−ΔΔ^CT approach. Specifically, the primers mentioned in Table 2 were used for the experiment.

**Table 2:** The information of qPCR primers

homo GAPDH	Forward	5’-GGTGTGAACCATGAGAAGTATGA-3’
	Reverse	5’-GAGTCCTTCCACGATACCAAAG-3’
homo CXCR7	Forward	5’-CTCTACACGCTCTCCTTCATTT-3’;
	Reverse	5’-GTGGTCTTGGCCTGGATATT-3’
homo ERK	Forward	5’-AGAGAACCCTGAGGGAGATAAA-3’
	Reverse	5’-CGATGGTTGGTGCTCGAATA-3’

### Western blotting

The cell lysis buffer (Beyotime, China) was used to harvest the EPCs proteins. Then, SDS-PAGE was run to isolate the extracted proteins, followed by transferring the proteins onto the PVDF membranes (Millipore, USA). Typically, the rabbit anti-phospho-ERK, rabbit anti-CXCR7, and rabbit anti-ERK antibodies (all 1:1000; Abcam, USA), and the mouse anti-GAPDH antibody (1:5000; Abbkine, China) were used. Later, the HRP-labeled anti-rabbit IgG and anti-mouse IgG (both 1:5000; Beyotime, China) were used for visualizing the proteins, and ECL chemiluminescence system (Tanon, China) was used for color development.

### Statistical analyses

The data were presented as mean ± standard deviation (SD). The SPSS 18.0 software was used for statistical analyses. The Kolmogorov-Smirnov test was used to study the distribution of the variables. The unpaired Student t-test or Welchs t-test (normal distribution) was employed to compare the two groups. The variance test (ANOVA) followed by the post-hoc test was used to evaluate the statistical significance of multiple groups of data with a parametric distribution. A difference of *P* < 0.05 indicated a statistical significance.

## Result

### Subject features

Differences in the baseline subject features were not statistically significant in normal controls compared to the CAD cases (Table 1). Figure S1 characterizes the late EPCs.

### Down-regulation of CXCR7/ERK signaling pathways decreased the CAD-derived EPCs function in vitro

**Fig. 1:**
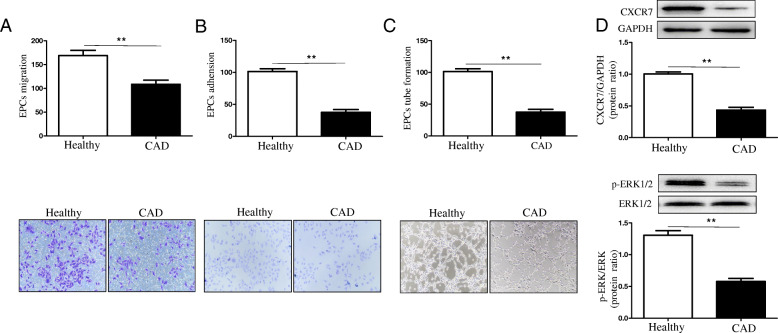
Down-regulation of CXCR7/ERK signaling decreased the CAD-derived EPCs functions in vitro. **a**: Quantitative analysis and typical images for EPCs migration (**P < 0.01 vs. normal EPCs; *n* = 3 in each group). **b**: Quantitative analysis and typical images for EPCs adhesion (**P < 0.01 vs. normal EPCs; *n* = 3 in each group). **c**: Quantitative analysis and typical images for the complete tube formation in EPCs (**P < 0.01 vs. normal EPCs; *n* = 3 in each group). **d**: Typical images and quantitative analysis on the protein levels of CXCR7 and p-ERK in cultured EPCs measured by Western Blotting (**P < 0.01 vs. normal EPCs; *n* = 3 in each group)

The late EPCs function in vitro was assessed to investigate the effect of CXCR7/ERK signaling pathways on EPCs dysfunction in CAD cases. Compared to the normal controls, the CAD-derived EPCs had declined migration and adhension capacity (Fig. 1a-b). The tube formation test was carried out to investigate the angiogenic potential of EPCs in vitro, thus determining EPCs vasculogenic function. The tube formation capacity of late EPCs in CAD cases declined consistently and remarkably compared to that of the healthy subjects (Fig. 1c). Meanwhile, the CXCR7 and p-ERK expression decreased within the CAD-derived EPCs when compared to that of the EPCs from normal controls, as verified by Western Blotting (Fig. 1d). Thus, it was speculated that CXCR7/ERK signaling pathways played a vital role in the normal EPCs function, and also the decreased CAD-EPCs vasculogenic ability may be associated with the down-regulated CXCR7/ERK signaling pathways to some extent.

### Shear stress up-regulated the CAD-derived EPCs proliferation, adhesion, and migration in vitro

**Fig. 2:**
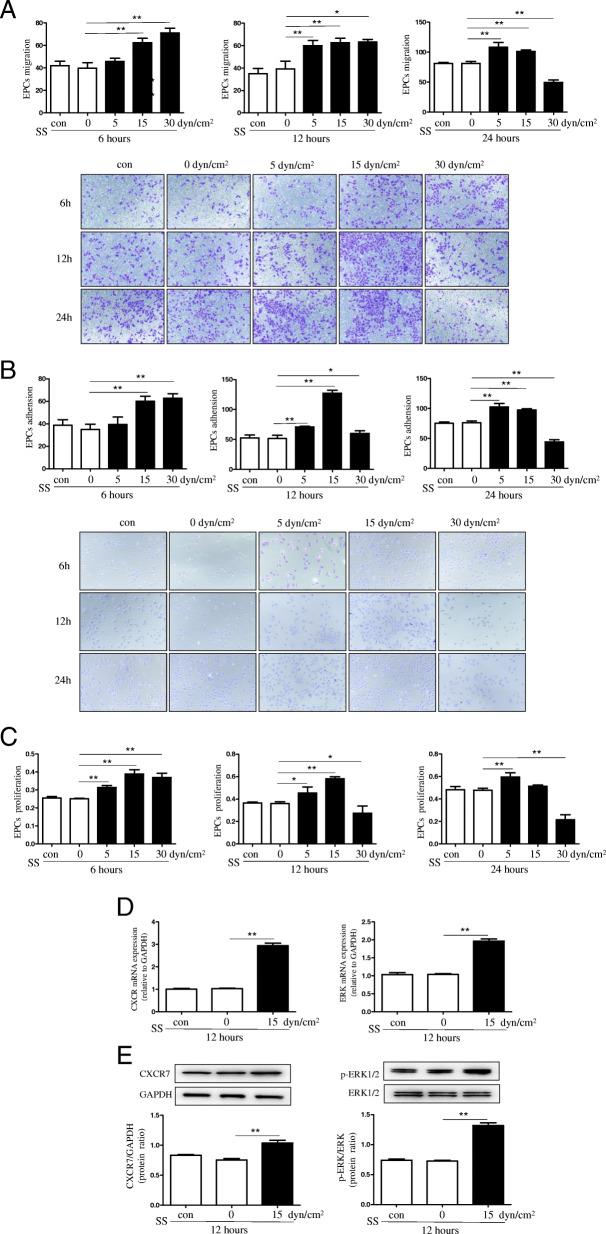
Shear stress enhanced the CAD-derived EPCs proliferation, adhesion, and migration capacities in vitro. **a**-**c**: CAD-derived EPCs were exposed to shear stress treatment for 6, 12, and 24 h, separately, at 0, 5, 15, and 30 dyn/cm^2^. **a**: Quantitative analysis and typical images for EPCs migration (**P < 0.01 vs. Static CAD-EPCs; *n* = 3 in each group). **b**: Quantitative analysis and typical images for EPCs adhesion (*p < 0.05/**p < 0.01vs. Static CAD-EPCs; *n* = 3 in each group). **c**: Quantitative analysis for EPCs proliferation (*p < 0.05/**p < 0.01vs. Static CAD-EPCs; *n* = 3 in each group). **d**: Quantitative analysis for the mRNA levels of CXCR7 and ERK in EPCs treated with or without 12 h of shear stress at 15 dyn/cm^2^ determined through Real-time PCR (*n* = 3 in each group). **e**: Quantitative analysis and typical images for the protein levels of CXCR7 and p-ERK/ERK in EPCs treated with or without 12 h of shear stress at 15 dyn/cm^2^ measured by Western Blotting (*p < 0.05/**p < 0.01vs. Static CAD-EPCs; *n* = 3 in each group)

To investigate the role of shear stress on the CAD-derived EPCs function in vitro, EPCs were exposed to 6, 12, and 24 h shear stress at 0, 5, 15, and 30 dyn/cm^2^, separately. The results suggested that shear stress significantly enhanced EPCs migration and adhesion capacity in CAD patients (Fig. 2a-b). Additionally, EPCs proliferation was promoted after being exposed to shear stress in vitro (Fig. 2c). Typically, laminar shear stress exposure at 15-dyn/cm^2^ for 12 h significantly enhanced the CAD-derived EPCs migration, adhesion, and proliferation activities. Thus, the relationship between the enhanced EPCs vasculogenic ability after being exposed to shear stress with the CXCR7/ERK signaling pathways was explored. We found that 12 h of shear stress exposure in EPCs at 15-dyn/cm^2^ markedly up-regulated the CXCR7 expression at mRNA (Fig. 2d) and protein (Fig. 2e) levels. Similarly, p-ERK levels (Fig. 2d-e) were remarkably up-regulated in the EPCs that underwent shear stress treatment compared towith those cultivated at static conditions.

### Shear stress exposure boosted the CAD-derived EPCs ability and affected apoptosis via the CXCR7/ERK signaling

**Fig. 3:**
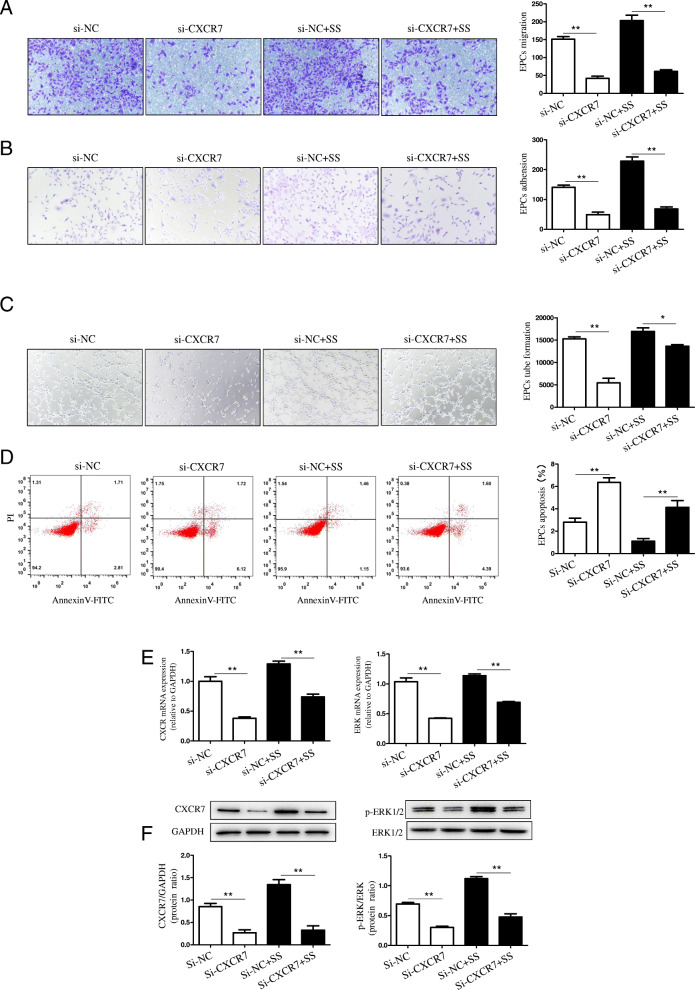
Blockade of CXCR7/ERK signaling mitigated the enhanced CAD-derived EPCs functions mediated by shear stress. **a**-**d**: The cultivated EPCs were subjected to scrambled-siRNA or Mission lentiviral CXCR7-siRNA infection for 48 h, and another 12 h of shear stress at 15 dyn/cm^2^. Then, migration, adhesion, tube formation, and apoptosis assays were carried out on these cells. **a**: Quantitative analysis and typical images for EPCs migration (**P < 0.01 vs. siRNA-transduced CAD-EPCs in the presence or absence of shear stress treatment; *n* = 3 in each group). **b**: Quantitative analysis and typical images for EPCs adhesion (**P < 0.01 vs. siRNA-transduced CAD-EPCs in the presence or absence of shear stress treatment; *n* = 3 in each group). **c**: Quantitative analysis and typical images for the complete tube formation of EPCs (**P < 0.01 vs. siRNA-transduced CAD-EPCs in the presence or absence of shear stress treatment; *n* = 3 in each group). **d**: Quantitative analysis and typical images for EPCs apoptosis (**P < 0.01 vs. siRNA-transduced CAD-EPCs with or without shear stress exposure; *n* = 3 in each group). **e**: Quantitative analysis for the CXCR7 and ERK mRNA levels in EPCs (**P < 0.01 vs. siRNA-transduced CAD-EPCs in the presence or absence of shear stress exposure; *n* = 3 in each group). **f**: Quantitative analysis and typical images for the protein levels of CXCR7 and p-ERK/ERK determined through Western Blotting (**P < 0.01 vs. siRNA-transduced CAD-EPCs with or without shear stress treatment; *n* = 3 in each group)

Subsequently, the effect of shear stress-mediated up-regulation of CXCR7/ERK signaling pathways in enhancing the EPCs functions was examined. According to our results, the lentiviral siRNA-induced CXCR7 silencing significantly attenuated the migratory (Fig. 3a), adhesive (Fig. 3b), and the tube forming (Fig. 3c) activities of CAD-derived EPCs on being exposed to shear stress in vitro. On the contrary, the transfection of lentiviral scrambled siRNA particles into EPCs made no difference to these functional factors. Further, the knockdown of CXCR7 declined apoptosis was mitigated by shear stress treatment in CAD patients (Fig. 3d). Overall, these findings revealed that exposure to shear stress boosted the CAD-derived EPCs vasculogenic ability through the CXCR7/ERK signaling pathways.

## Discussion

The result of this study were as follows: (1) the migration, adhesion and tube formation activities of CAD-derived EPCs in vitro, remarkably decreased compared to those of the healthy subjects; (2) diminishing the CXCR7/ERK signaling pathways impaired the CAD-derived EPCs function in vitro; and (3) exposure to shear stress mitigated the impaired EPCs function in the CAD patients by augmenting the CXCR7/ERK pathways. Overall, our study demonstrated that the CXCR7/ERK signaling pathways played an essential role in the CAD-derived EPCs function, and the shear stress exposure in vitro may be a favorable method to enhance the EPCs function.

The circulating EPCs are of great importance in maintaining endothelial integrity after endothelial injury, and CAD is related to decrease EPCs number and function in the human body [12, 28–30]. In this study, the CAD-derived EPCs had markedly decreased abilities compared to those collected from the normal controls, suggesting that CAD decreased the endogenous vasculogenic abilities of EPCs. Emerging evidence indicates that CXCR7 plays an important role in regulating multiple cellular functions [20–22, 31]. Moreover, in the previous studies, CXCR7 regulates the EPCs proliferating, adhesive and vasculogenic activities in vitro [32, 33]. The CXCR7 signaling was closely associated with EPCs functions, and thus, we assumed that the dysregulated CXCR7 signaling may be associated with EPCs functional impairment in the CAD patients. This study suggested that the CXCR7 level was remarkably decreased in CAD-derived EPCs compared to those in normal controls, as previously reported [23]. ERK is a critical molecular signaling pathway involved in the survival, differentiation, and proliferation of various cells. Therefore, we assumed that the ERK signaling pathway participated in regulating the EPCs function mediated by CXCR7 [34–36]. For a better understanding of the relationship between CXCR7 and ERK, the phosphorylated ERK (p-ERK) level was determined, which suggested that p-ERK level remarkably decreased in CAD-derived EPCs compared to that of the EPCs from healthy subjects. According to these results, the CXCR7/ERK signaling pathways play a vital role in the EPCs function. Therefore, a novel approach that enhances the CXCR7/ERK signaling pathways can also potentially enhance the EPCs vasculogenic activities while reversing the vascular endothelial integrity and homeostasis in CAD patients.

Previous studies report that shear stress plays an important role in modulating the EPCs structure and function, which indicates that it as a vital non-pharmacologic approach in regulating EPCs function [26, 37, 38]. Nonetheless, the role of shear stress in the level of CXCR7 in EPCs, as well as its correlation with endothelial protection in the CAD cases, remains unclear. This study speculated that shear stress possibly facilitated the CXCR7/ERK signaling pathways to promote EPCs vasculogenic activities. To test this speculation, the role of shear stress in the CAD-derived EPCs proliferation, migration, and adhesion capacities in vitro was monitored, and according to our results, laminar shear stress facilitated the proliferation, adhesion, and migration of CAD-derived EPCs in vitro. Also, the shear stress upregulated the CXCR7 level and the ERK phosphorylation in EPCs of the CAD patients. Further, such augmentation of shear stress-induced CXCR7/ERK signaling pathways in CAD-derived EPCs conformed to the increased migratory, adhesive, and the proliferative capacities in vitro. However, the shear stress-mediated enhanced EPCs function was mitigated via CXCR7-siRNA. The above results indicate that shear stress may be favorable for modifying the CAD-derived EPCs biological phenotype.

Our results display a strong clinical significance. The dysfunction of EPCs represents the starting step, as well as a primary factor in the dismal angiogenesis of CAD [39, 40]. Clearly, our findings show that the dysfunctional properties of EPCs are associated with CAD, suggesting that CAD negatively affects the maintenance of vascular homeostasis. Typically, identifying that CAD decreases the CXCR7/ERK signaling pathways may offer molecular targets in developing a treatment to enhance the endogenous EPCs capacity. Our findings also provide evidence that shear stress may restore CXCR7 signaling and promote EPCs capacity. This presents a vital cell-based treatment to enhance EPCs function and improve vascular repairability in CAD cases. Moreover, our study also demonstrated that the blockade of CXCR7 resulted in the down-regulation of the ERK signaling pathway after the shear stress treatment, implying that the ERK pathway is involved in the angiogenesis of CAD-derived EPCs mediated by the shear stress. All in all, our work sheds more light on EPCs-related dysfunction in CAD patients offering a new strategy in the prevention and treatment of CAD.

However, several limitations are noted in this study. Firstly, only the role of shear stress in regulating the CXCR7/ERK signaling pathways, and its association with the repairability of EPCs, in vitro was examined in this study. But, an in vivo study is warranted to examine the EPCs vasculogenic ability. Secondly, this study indicated that the abnormal CXCR7 signaling in EPCs participated in the impaired CAD-derived EPCs function but, the underlying mechanism of the reduced CXCR7 level has not been well understood yet. Thirdly, this work illustrated the effects of shear stress on the phosphorylation of CXCR7/ERK signaling pathways in the EPCs, yet the precise mechanism was not clarified.

## Conclusion

In summary, the results show that the shear stress enhances CAD-derived EPCs vasculogenic abilities through boosting the activation of the CXCR7/ERK signaling pathways to some extent. Based on our findings, the shear stress effect on regulating the CXCR7/ERK signaling pathways can be an underlying treatment target to repair the EPCs function in the CAD patients.

## Supplementary information

**Additional file 1: Figure S1.** Phenotypic characterization of L-EPCs.

**Additional file 2: Figure S2.** Analysis of CXCR7 and p-ERK expression in normal controls and CAD-EPCs.

**Additional file 3: Figure S3.**The effect of shear stress on CXCR7 and p-ERK expression in CAD-EPCs.

**Additional file 4: Figure S4.** Knockdown of CXCR7 significantly attenuated the p-ERK expression of CAD-derived EPCs following shear stress in vitro.

## Data Availability

All data generated or analyzed during this study are included in this published article and its supplementary information files.

## Abbreviations

EPCs: Endothelial progenitor cells
CAD: Coronary artery disease;
CXCR7: CXC chemokine receptor 7
ERK: Extracellular signal-regulated kinase
CVDs: Cardiovascular diseases
SDF-1: Stromal cells-derived factor 1
BMI: Body mass index
HR: Heart rate
PBMCs: Peripheral blood mononuclear cells
FBS: Fetal bovine serum
EGM-2: Endothelial cell growth medium-2
siRNA: Small interfering RNA
qRT-PCR: Quantitative reverse transcription PCR
GAPDH: Glyceraldehyde-3-phosphate dehydrogenase
FACS: Fluorescence activated cell sorting
CD31: Platelet endothelial cell adhesion molecule
Tie-2: Tyrosine-protein kinase receptor-2
vVF: Von Willebrand factor.
