# Supplementary figures and images for: Shear stress improves the endothelial progenitor cell function via the CXCR7/ERK pathway axis in the coronary artery disease cases
Source: BMC Cardiovasc Disord. 2020 Sep 7;20:403. doi: 10.1186/s12872-020-01681-0 (PMC7487552; doi:10.1186/s12872-020-01681-0)

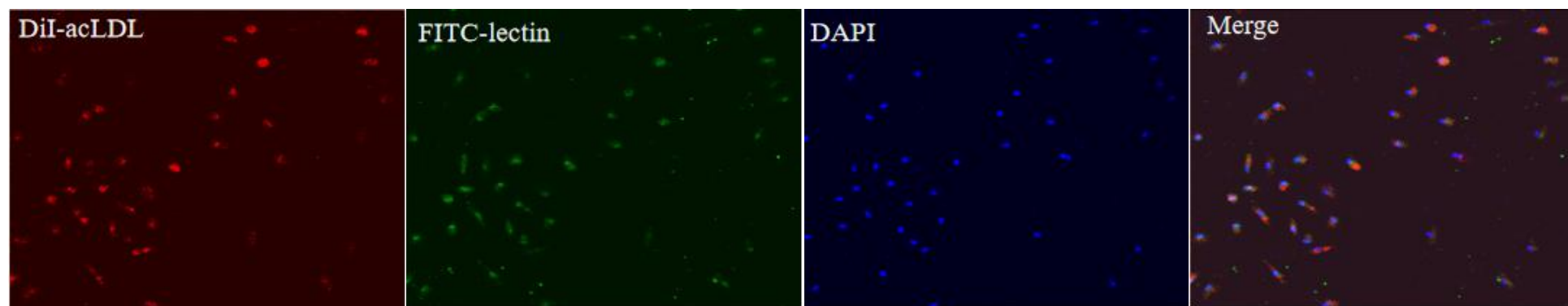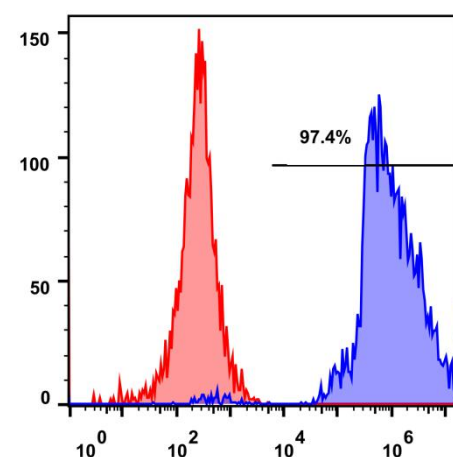

CD31 FITC

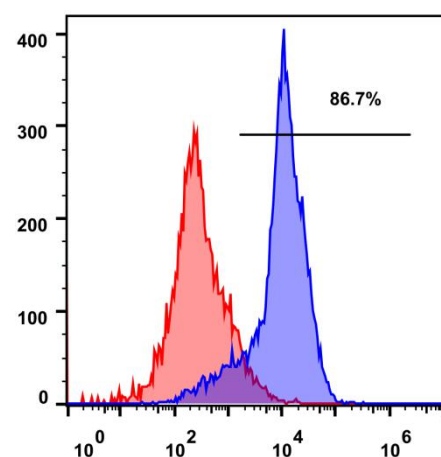

Tie-2 APC

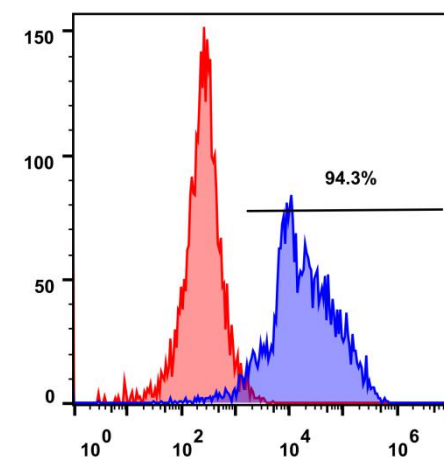

vWF FITC

Supplement: Supplementary file 1 — Additional file 1: Figure S1. Phenotypic characterization of L-EPCs. [file 12872_2020_1681_MOESM1_ESM.pdf]

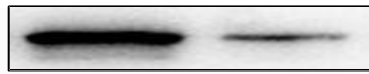

Fig1.D

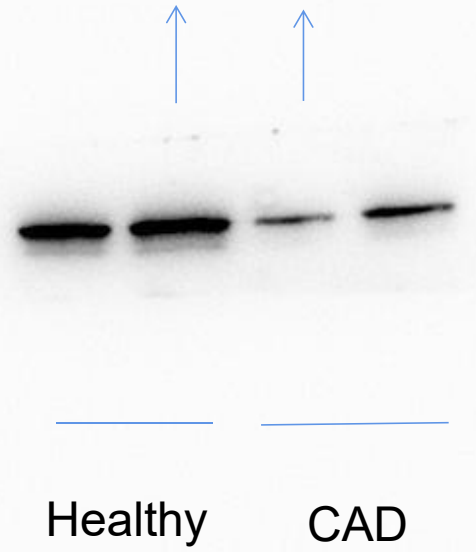

CXCR7

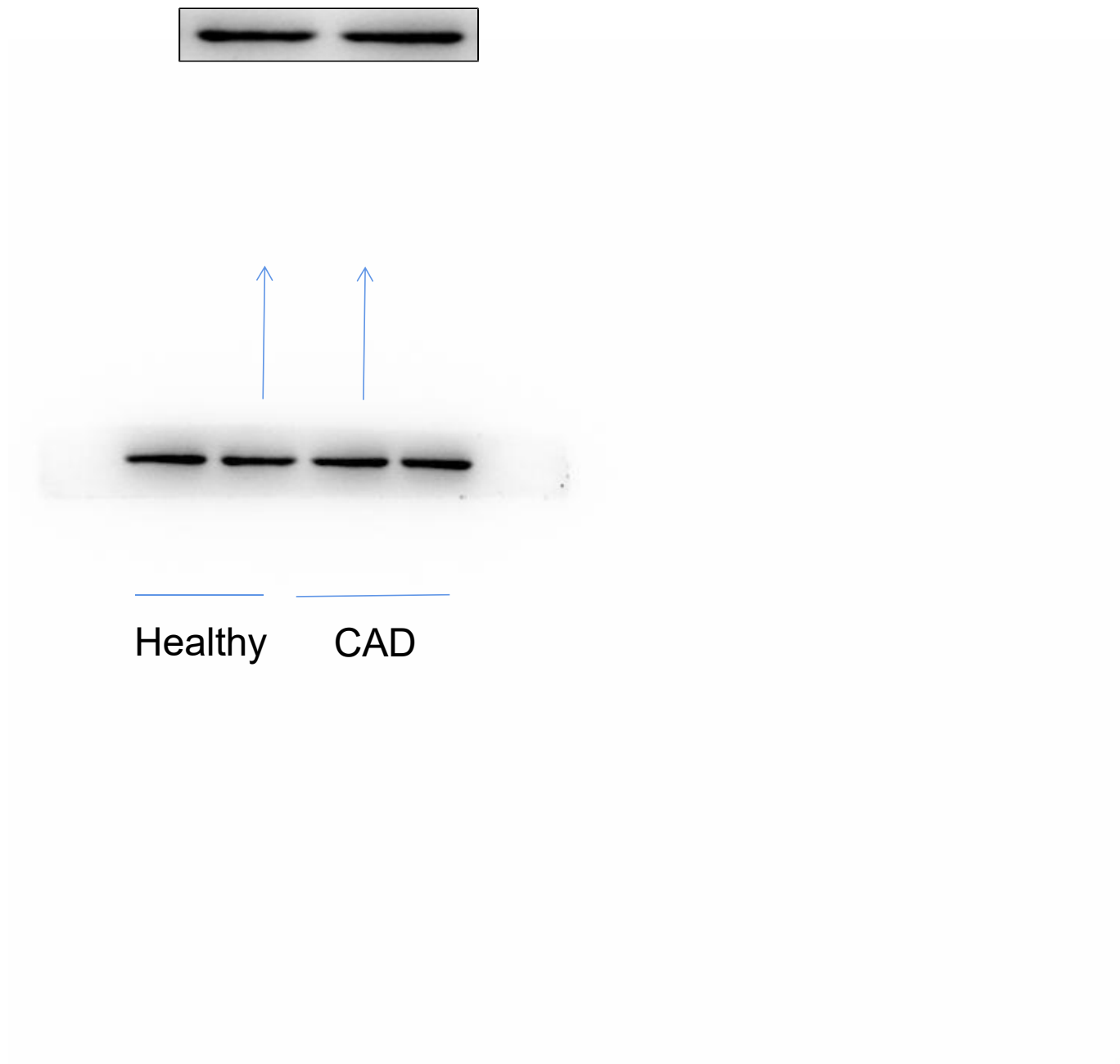

Fig1.D

GAPDH

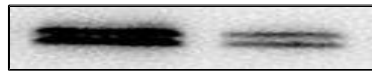

Fig1.D

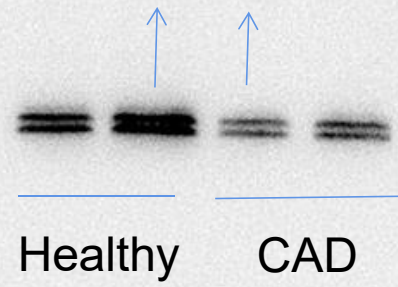

p-ERK 1/2

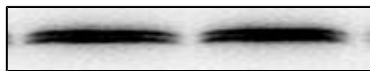

Fig1.D

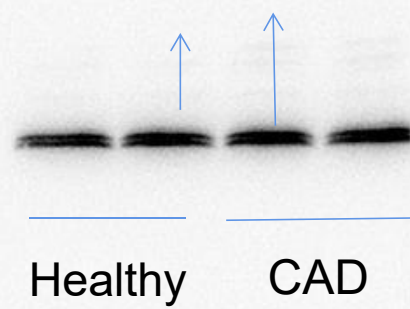

ERK 1/2

Supplement: Supplementary file 2 — Additional file 2: Figure S2. Analysis of CXCR7 and p-ERK expression in normal controls and CAD-EPCs. [file 12872_2020_1681_MOESM2_ESM.pdf]
