# Supplementary material for: Shear stress improves the endothelial progenitor cell function via the CXCR7/ERK pathway axis in the coronary artery disease cases
Source: BMC Cardiovasc Disord. 2020 Sep 7;20:403. doi: 10.1186/s12872-020-01681-0 (PMC7487552; doi:10.1186/s12872-020-01681-0)
Supplement: Supplementary file 3 — Additional file 3: Figure S3.The effect of shear stress on CXCR7 and p-ERK expression in CAD-EPCs. [file 12872_2020_1681_MOESM3_ESM.pdf]

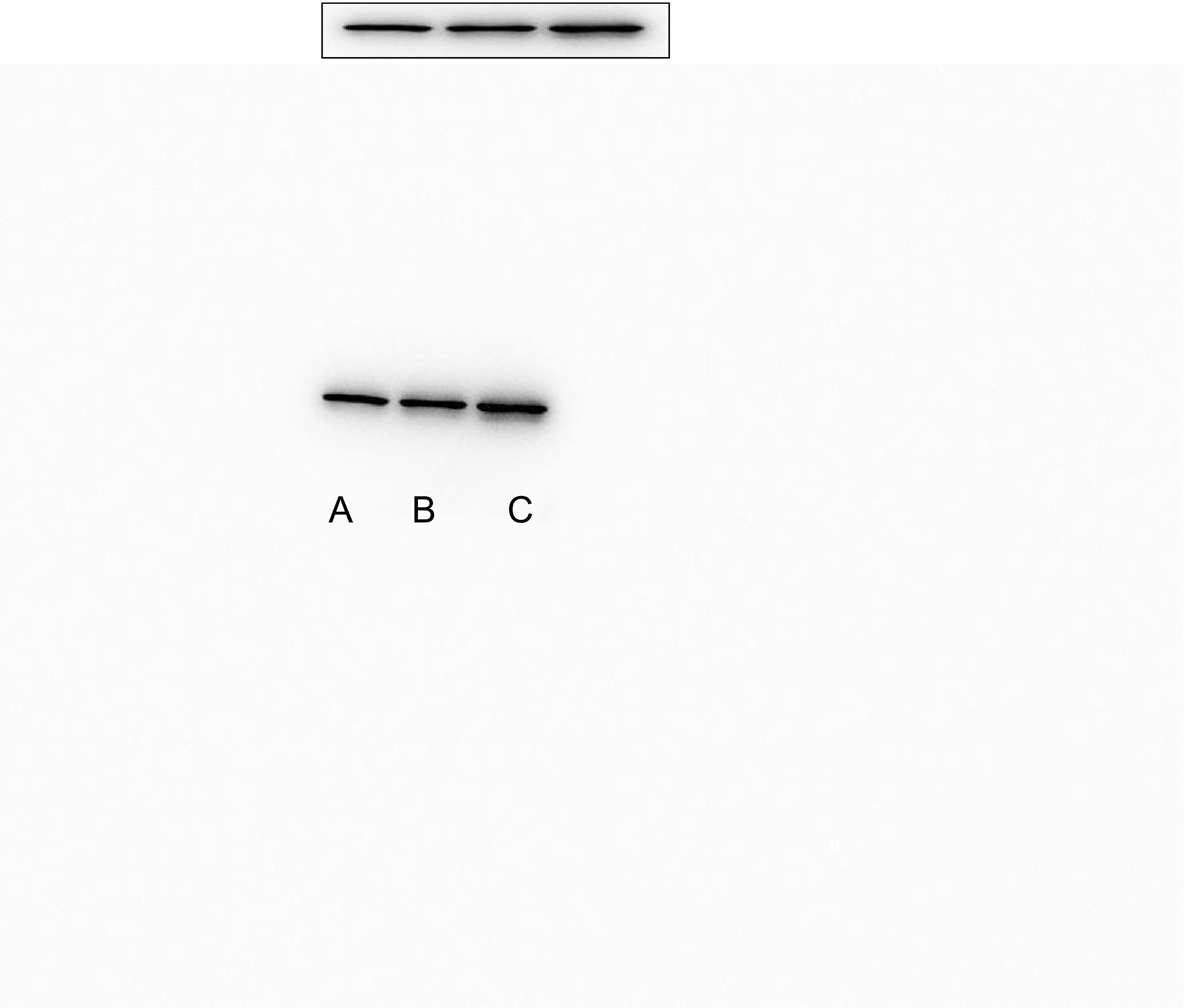

Fig2.D

CXCR7

A: control
B： 0 dyn/cm2
C: 15 dyn/cm2 for 12hours

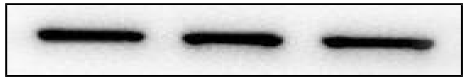

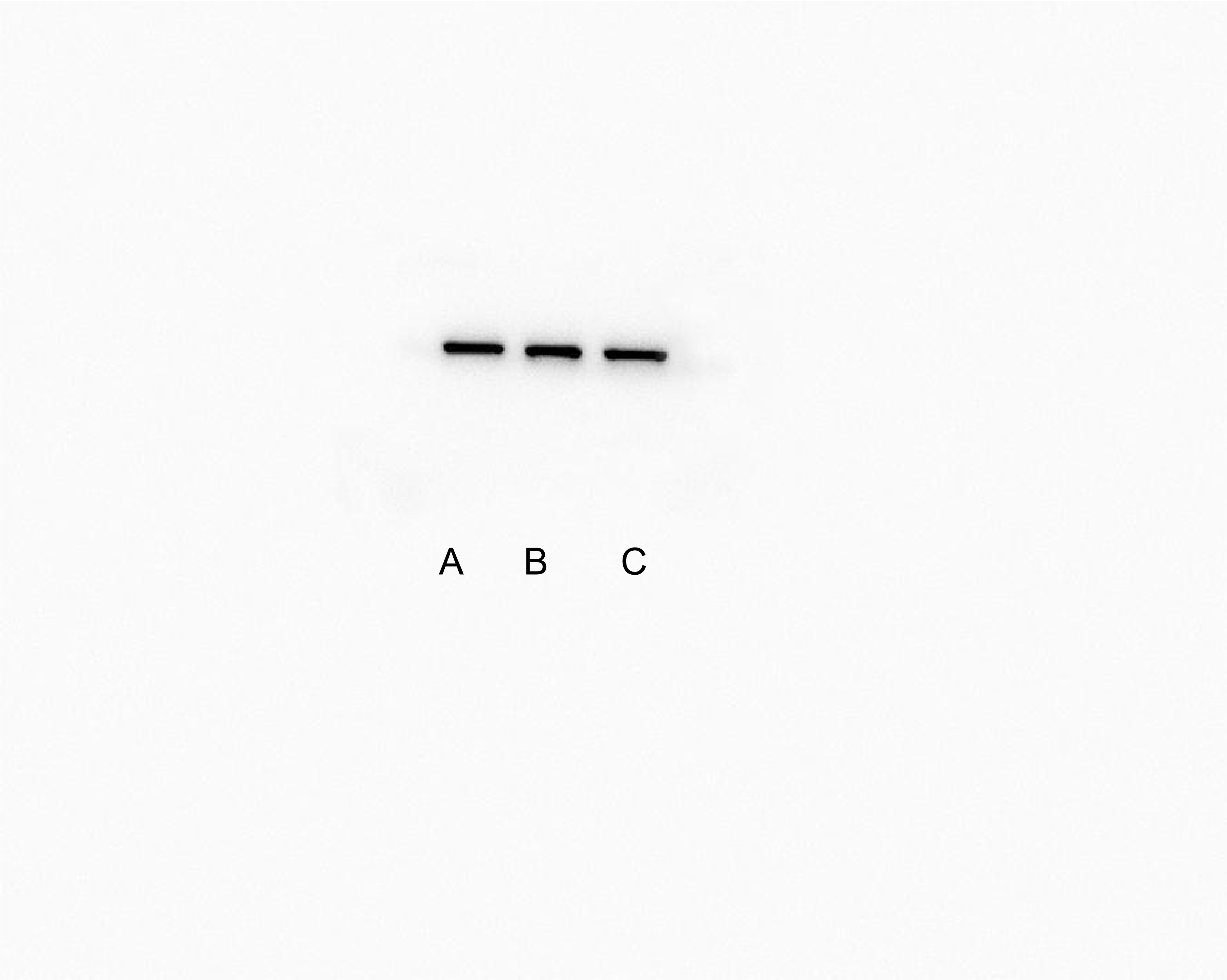

Fig2.D

GAPDH

A: control
B： 0 dyn/cm2
C: 15 dyn/cm2 for 12hours

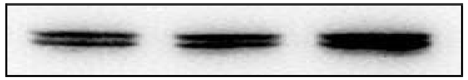

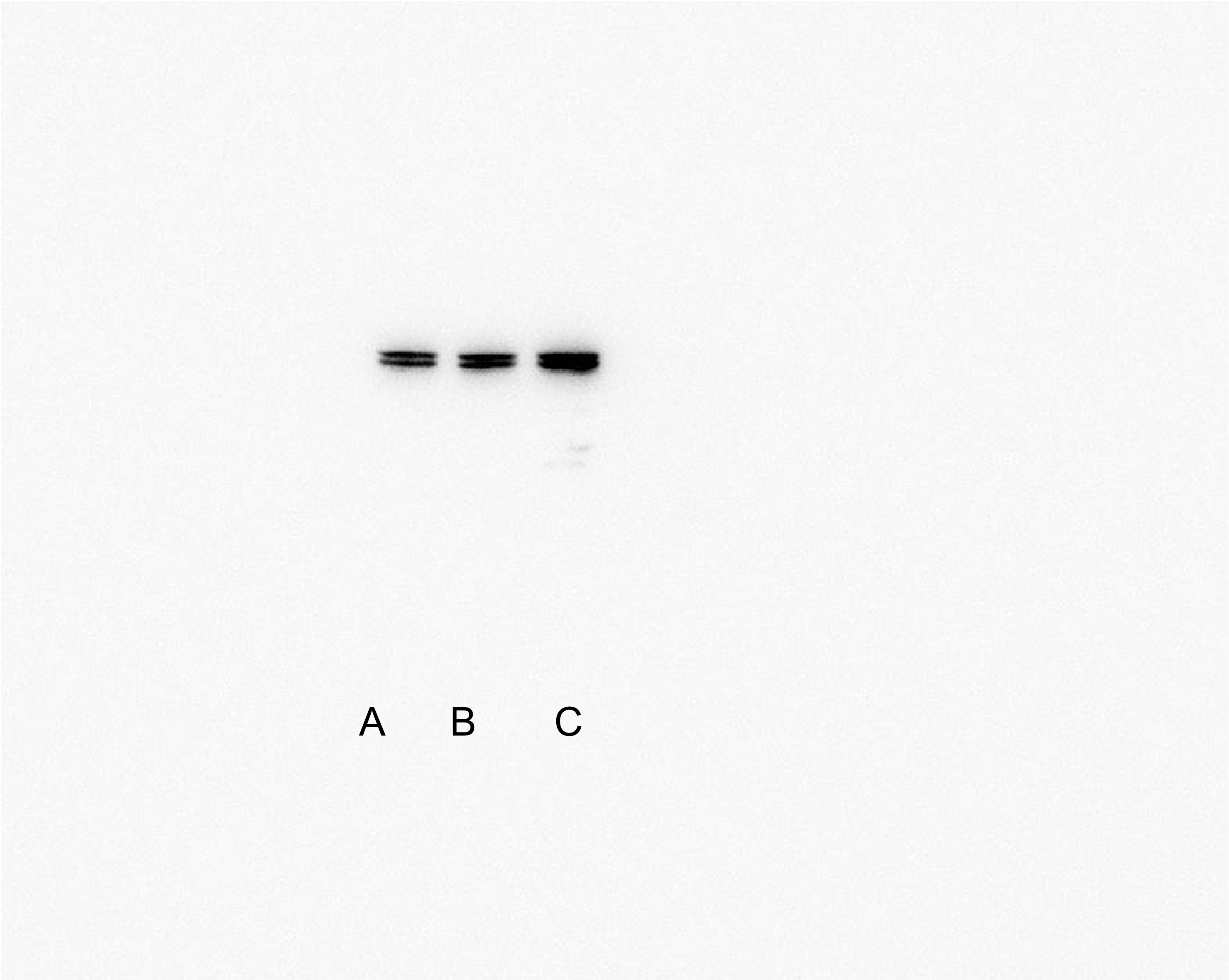

Fig2.D

p-ERK 1/2

A: control
B： 0 dyn/cm2
C: 15 dyn/cm2 for 12hours

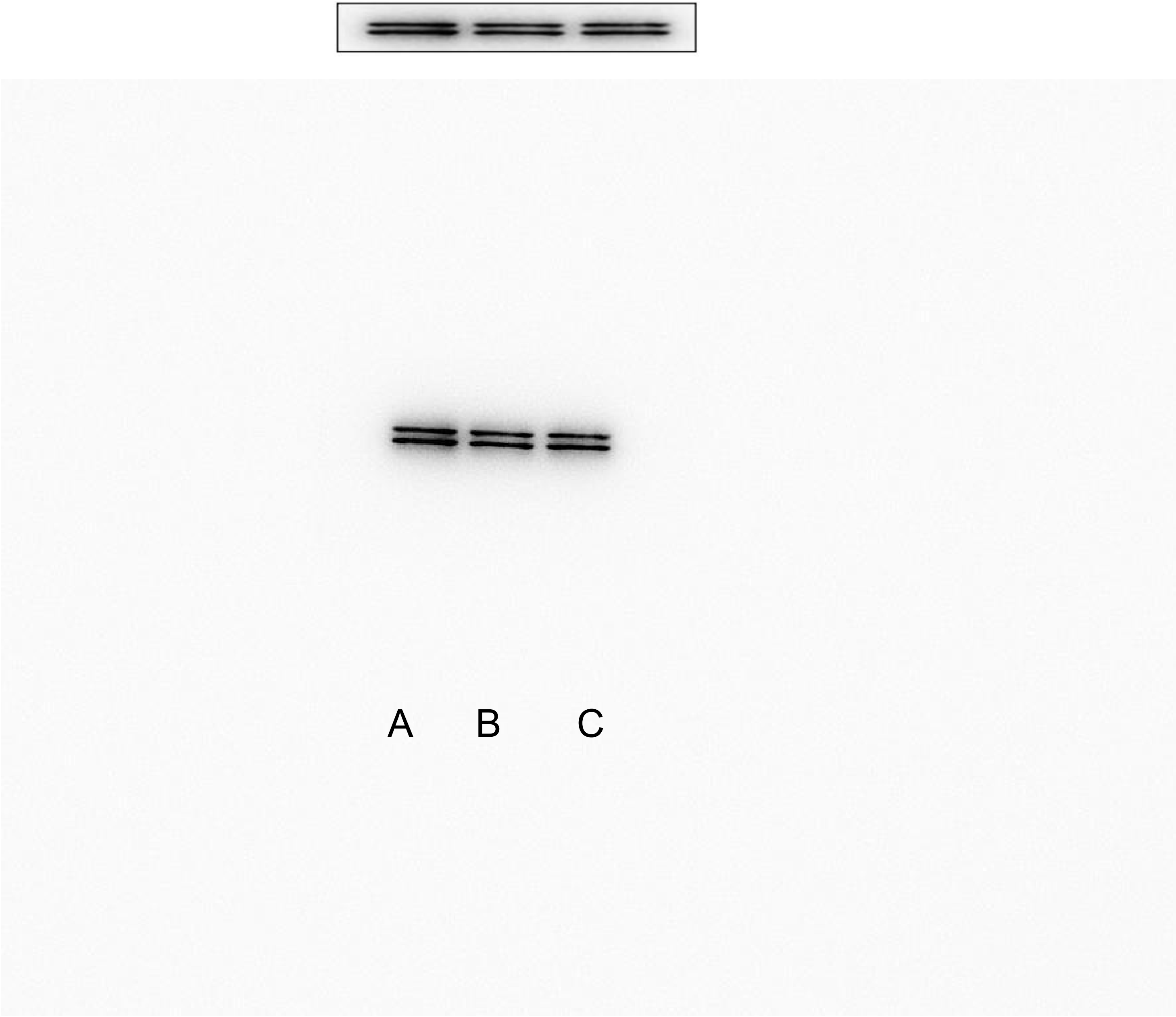

Fig2.D

ERK 1/2

A: control

B： 0 dyn/cm2

C: 15 dyn/cm2 for 12hours
