# Supplementary material for: Shear stress improves the endothelial progenitor cell function via the CXCR7/ERK pathway axis in the coronary artery disease cases
Source: BMC Cardiovasc Disord. 2020 Sep 7;20:403. doi: 10.1186/s12872-020-01681-0 (PMC7487552; doi:10.1186/s12872-020-01681-0)
Supplement: Supplementary file 4 — Additional file 4: Figure S4. Knockdown of CXCR7 significantly attenuated the p-ERK expression of CAD-derived EPCs following shear stress in vitro. [file 12872_2020_1681_MOESM4_ESM.pdf]

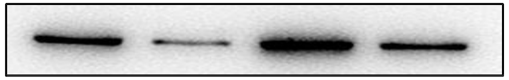

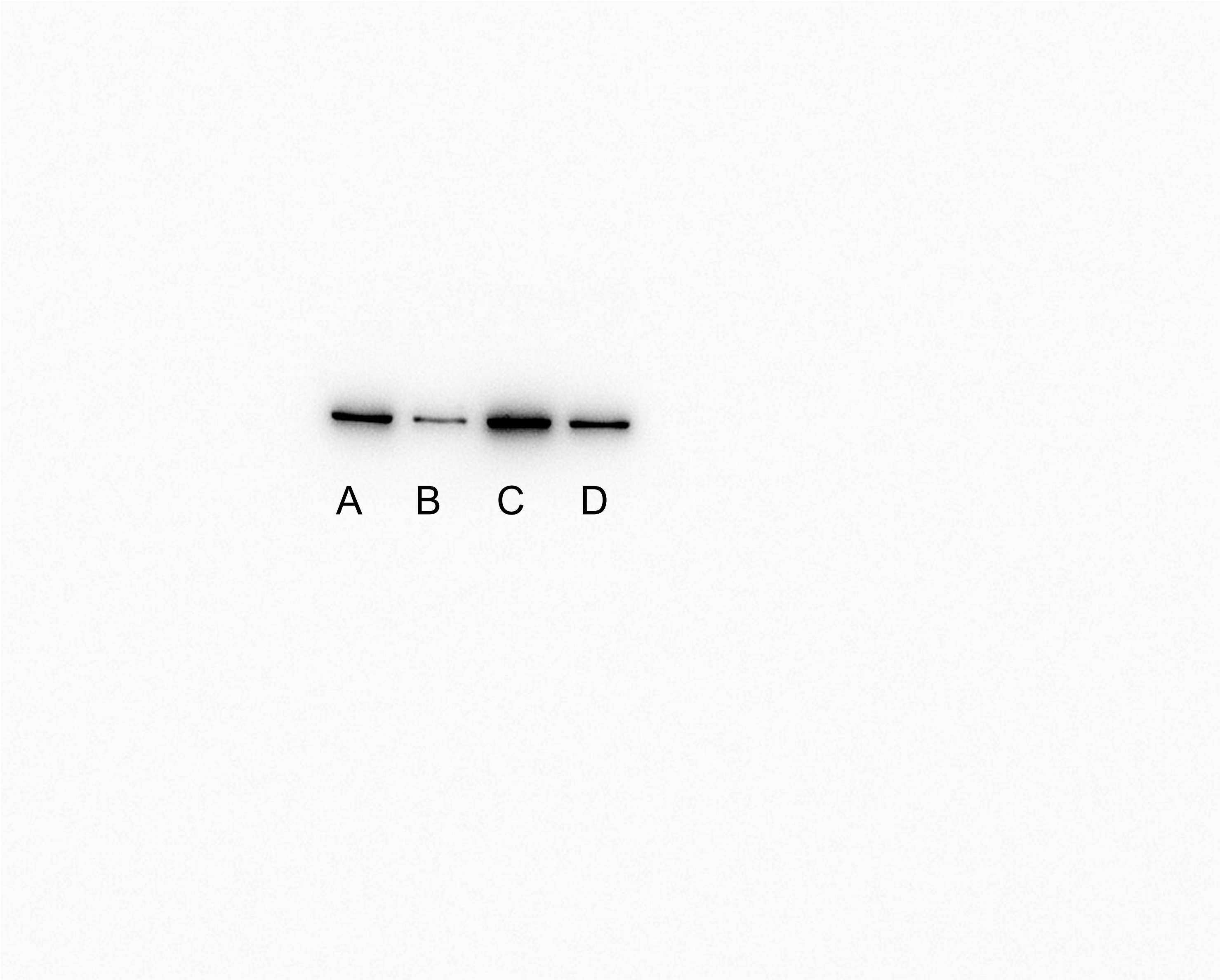

Fig3.F

CXCR7

A: si-NC

B： si-CXCR7

C: si-NC+shear stress for 12 hours

D: si-CXCR7+shear stress for 12 hours

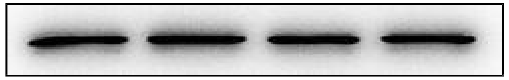

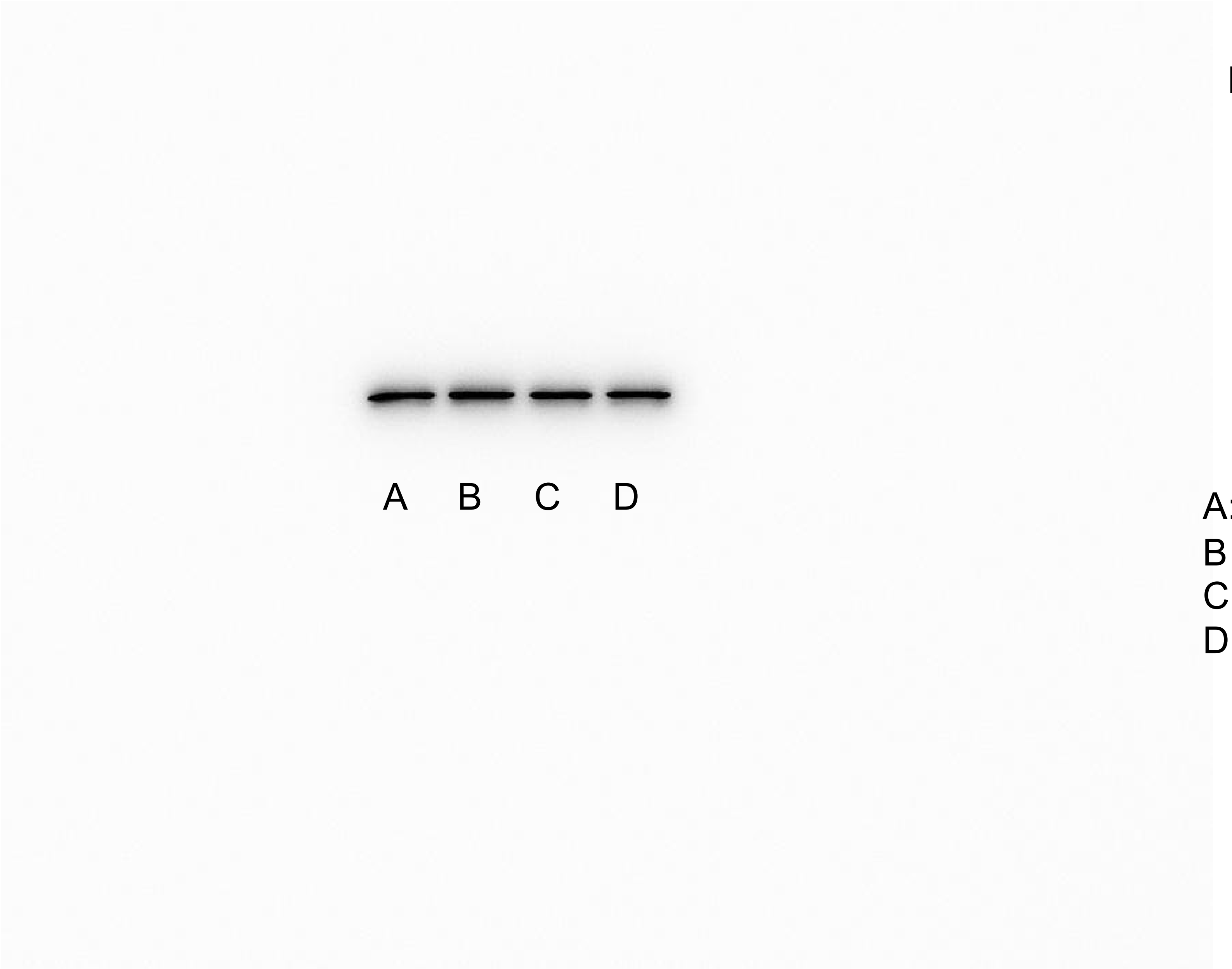

Fig3.F

GAPDH

A: si-NC

B： si-CXCR7

C: si-NC+shear stress for 12 hours

D: si-CXCR7+shear stress for 12 hours

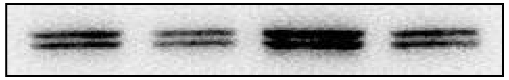

Fig3.F

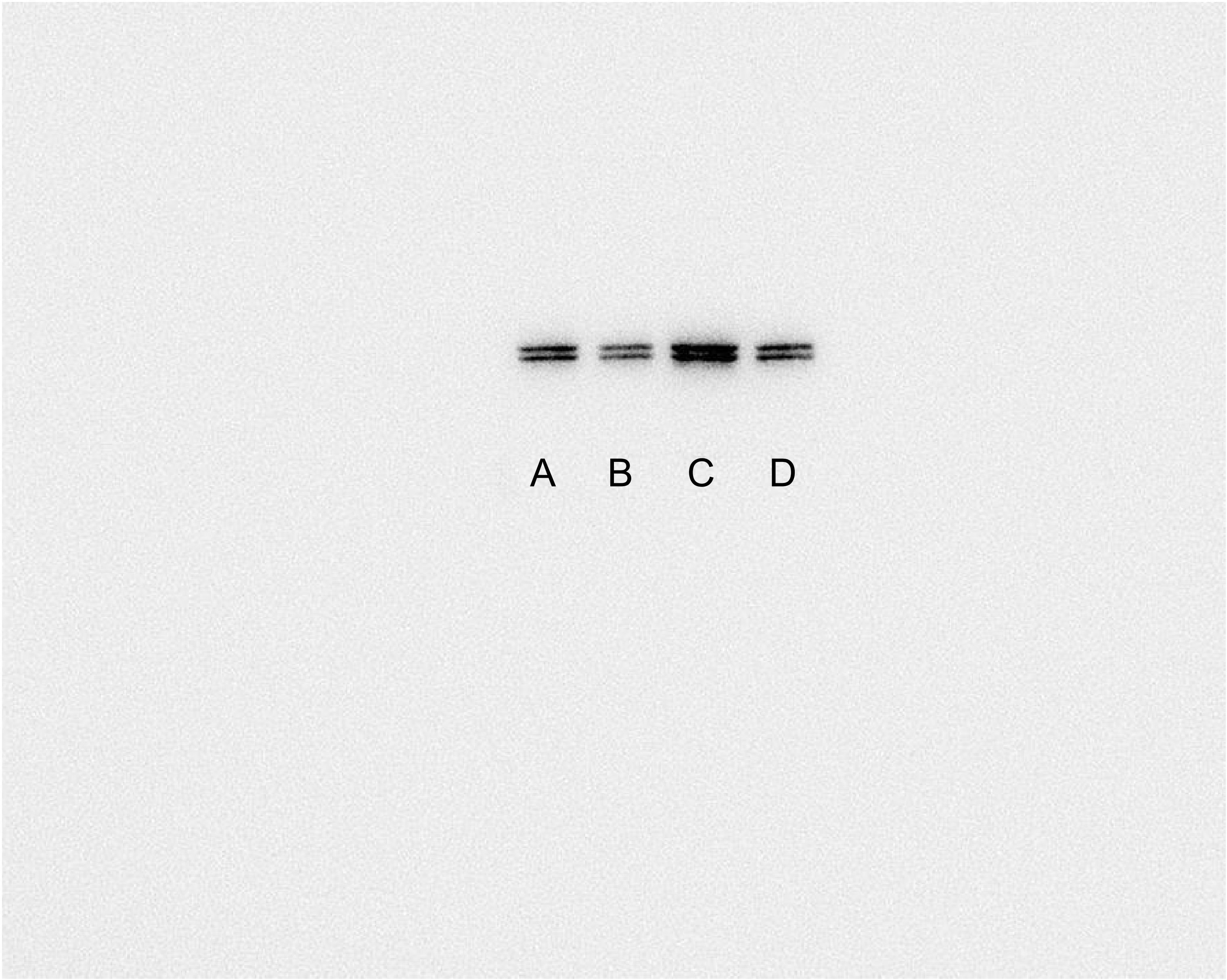

p-ERK 1/2

A: si-NC
B： si-CXCR7
C: si-NC+shear stress for 12 hours
D: si-CXCR7+shear stress for 12 hours

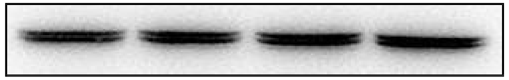

Fig3.F

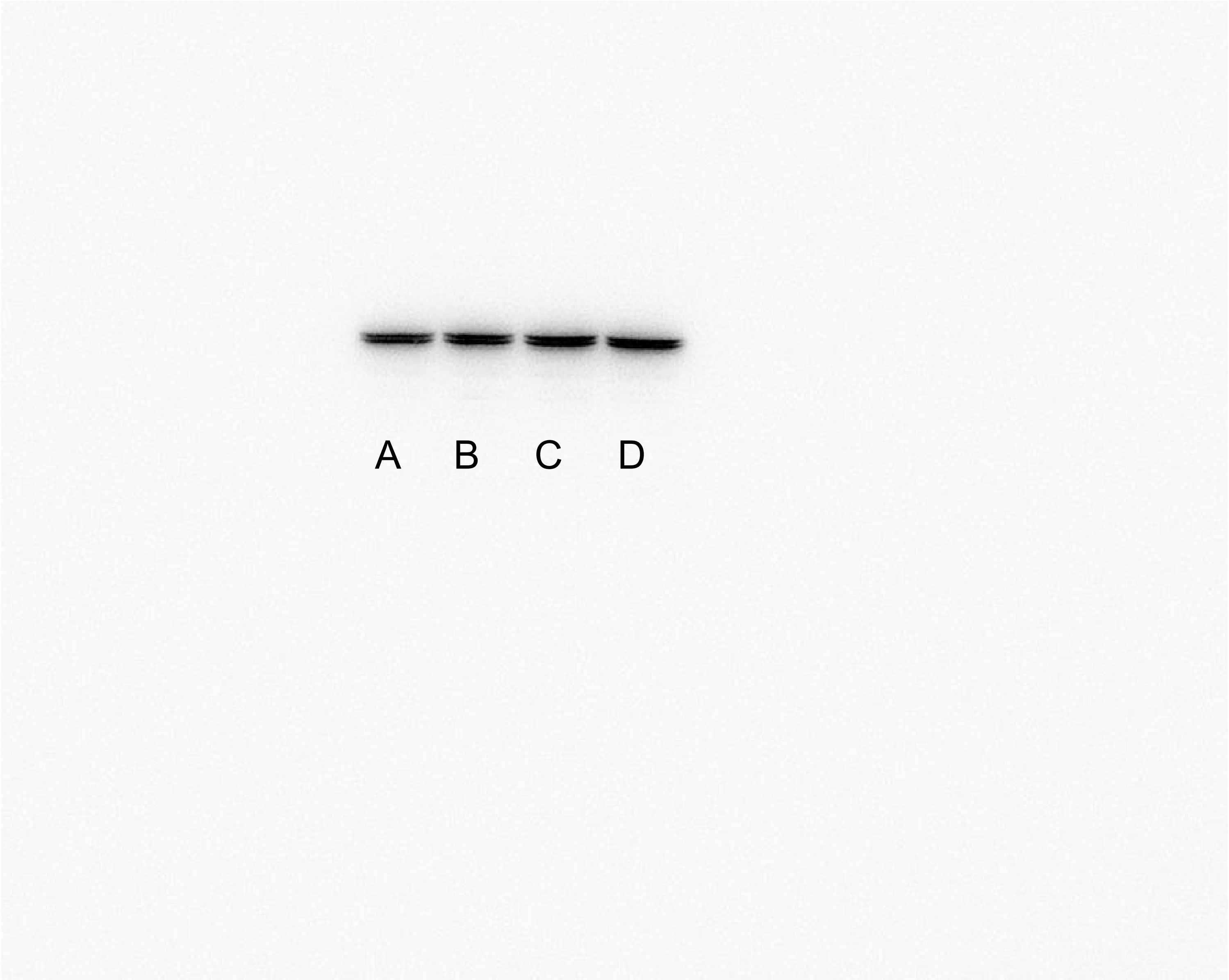

ERK 1/2

A: si-NC
B： si-CXCR7
C: si-NC+shear stress for 12 hours
D: si-CXCR7+shear stress for 12 hours
